# Tumor-intrinsic execution of interferon-γ-associated immunity in bladder cancer: checkpoints in signaling, recruitment, and recognition

**DOI:** 10.3389/fimmu.2026.1979524

**Published:** 2026-09-25

**Authors:** Jingjing Lu, Yichun Jiang, Yanshan Jin, Yi Zhang

**Affiliations:** 1 Department of Reproductive Medicine, The Affiliated People’s Hospital of Ningbo University, Ningbo, China; 2 Department of Urology, The Affiliated People’s Hospital of Ningbo University, Ningbo, China

**Keywords:** antigen presentation, bacillus Calmette–Guérin, bladder cancer, CXCL10, CXCL9, immune checkpoint blockade, interferon-γ, tumor immunity

## Abstract

Interferon-γ (IFN-γ) is a central mediator of antitumor immunity in bladder cancer, but an IFN-γ-rich tumor microenvironment does not necessarily translate into tumor control. Tissue-level inflammatory signatures capture overall immune activity and cannot determine whether malignant urothelial cells remain responsive to immune-derived IFN-γ or preserve the downstream functions required for immune elimination. This Mini Review examines tumor-intrinsic IFN-γ-associated immunity at three functional checkpoints: proximal IFN-γ sensing and signal transduction, chemokine-mediated effector-cell recruitment, and antigen-presentation-dependent tumor recognition. Bladder cancer models show that IFNGR–JAK–STAT1 responsiveness can be lost or attenuated, while separate tumor-intrinsic mechanisms can restrict CXCL9/CXCL10-dependent T-cell recruitment without a demonstrated proximal signaling defect. Antigen presentation forms a further checkpoint, with HLA-I and antigen-processing machinery altered through transcriptional, treatment-associated, and post-translational mechanisms. These mechanisms do not constitute a single linear IFN-γ resistance pathway. They instead mark distinct points at which inflammatory pressure can become uncoupled from tumor elimination. IFN-γ-responsive programs are also shaped by treatment context and signaling duration, as illustrated by CIITA/MHC-II biology and prolonged interferon signaling. Resolving these processes at malignant-cell, spatial, and longitudinal levels may improve the interpretation of BCG and checkpoint-blockade resistance and support more selective combination strategies.

## Introduction

Bladder cancer has been treated with immunotherapy for decades. Intravesical bacillus Calmette–Guérin (BCG) remains a standard treatment for high-risk non-muscle-invasive bladder cancer, while antibodies targeting programmed cell death protein 1 (PD-1) or its ligand PD-L1 are used in several settings of urothelial carcinoma. Clinical benefit, however, varies substantially among tumors, and immune-cell abundance alone does not explain this heterogeneity.

Interferon-γ (IFN-γ), produced largely by activated T cells and natural killer cells, is an important signal in the interaction between immune cells and malignant urothelium. In differentiated human urothelial cells, IFN-γ induces CXCL9, CXCL10, and CXCL11 together with genes involved in antigen processing and presentation, including B2M, TAP1, TAP2, CD74, and CIITA (1). In muscle-invasive bladder cancer, inflammatory tumors are likewise enriched for IFN-γ-responsive chemokines, antigen-processing pathways, and MHC-related programs (2). CD80 and CD86 are not induced to the same extent in differentiated urothelium, so an IFN-γ-responsive urothelial cell should not be considered equivalent to a conventional professional antigen-presenting cell (1).

Clinical datasets show a related pattern. In a meta-analysis of six advanced bladder cancer ICI cohorts, responders had higher CXCL9, CXCL10, and IFNG expression, greater immune-cell abundance, and enrichment of antigen-processing and HLA-related signatures (3). CheckMate 275 associated an IFN-γ gene-expression signature and CXCL9/CXCL10-rich tumors with nivolumab activity, while responses occurred across PD-L1 strata (4). Exploratory analyses from KEYNOTE-045 and KEYNOTE-052 also linked T-cell-inflamed programs with pembrolizumab outcomes, although individual biomarkers performed differently across settings (5). A score integrating T-cell and stromal features similarly suggests that immune infiltration is more informative when interpreted in tissue context (6). These are tissue-level associations and do not establish malignant-cell IFN-γ responsiveness.

Inflammation can also coexist with resistance through mechanisms that are largely independent of tumor-cell IFN-γ sensing. In metastatic urothelial carcinoma, a fibroblast TGF-β response signature was associated with non-response specifically in immune-excluded tumors, in which CD8+ T cells accumulated in desmoplastic regions rather than contacting malignant cells (7). Inflammatory activity, spatial access, and tumor-cell responsiveness are therefore related but non-equivalent features of the tumor microenvironment.

Here, the focus is the malignant urothelial cell: whether and how it executes immune programs associated with IFN-γ exposure. We use three functional descriptors—signal competence, recruitment competence, and recognition competence—to organize the available evidence. They are not presented as a validated molecular classification, and not every molecule discussed below is part of canonical IFN-γ signaling. The framework is intended to identify where tumor-intrinsic execution may become uncoupled from an otherwise inflammatory immune environment, rather than to redefine the canonical IFN-γ pathway.

## Tumor-cell responsiveness as a functional bottleneck in bladder cancer immunity

BCG experiments provide direct evidence that tumor-cell IFN-γ responsiveness can matter for antitumor immunity. In the orthotopic MB49 model, deletion of IFNGR from tumor cells markedly impaired BCG-mediated tumor control. IFNGR-deficient tumors could also escape previously established BCG-induced tumor immunity: four of five BCG-survivor mice failed to reject IFNGR-deficient MB49 tumors, whereas wild-type tumors were rejected (8). Tumor-cell MHC-II expression was not required for the same response, arguing against an explanation based solely on IFN-γ-induced MHC-II (8).

In this model, immune activation alone was therefore insufficient when malignant cells could not receive the IFN-γ signal. The finding should not be extended to every form of bladder cancer immunotherapy, because the direct evidence comes from BCG-treated MB49 tumors.

For the purposes of this Review, the downstream biology is considered at three levels. Signal competence refers to IFNGR–JAK–STAT1 signaling within the malignant cell. Recruitment competence refers to the production of chemotactic signals, particularly the CXCR3 ligands CXCL9, CXCL10, and CXCL11. Recognition competence refers to the HLA-I and antigen-processing machinery required for cytotoxic T-cell recognition.

These levels are separable. Chemokine production is regulated transcriptionally and post-transcriptionally beyond IFNGR signaling, whereas HLA-I expression can change through mechanisms that do not require loss of proximal STAT1 activation. The framework thus describes functional checkpoints rather than a single biochemical cascade. These three functional checkpoints and the major tumor-intrinsic mechanisms discussed in this Review are summarized in **Figure 1**.

**Figure 1:**
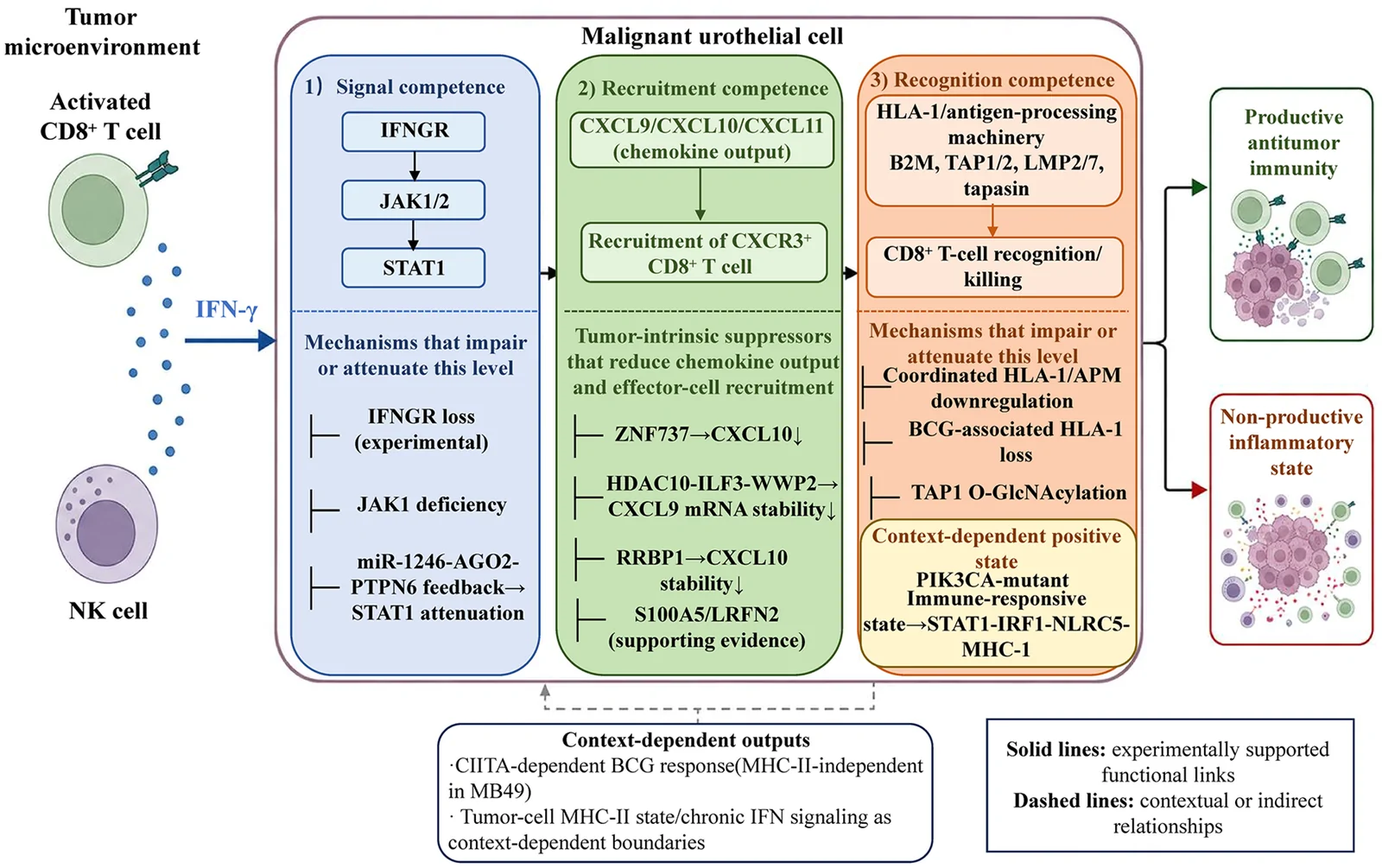
Tumor-intrinsic checkpoints linking IFN-γ-associated inflammation to antitumor immunity in bladder cancer. Immune-derived IFN-γ signals through IFNGR–JAK–STAT1 in malignant urothelial cells. Productive immune execution requires preserved proximal signaling together with two downstream functions: CXCL9/CXCL10/CXCL11-associated recruitment of CXCR3+ effector cells and HLA-I/APM-dependent tumor recognition. IFNGR dysfunction, JAK1 deficiency, and the miR-1246–AGO2–PTPN6 circuit can attenuate proximal IFN-γ responsiveness. ZNF737, HDAC10–ILF3–WWP2, RRBP1, S100A5, and LRFN2 regulate inflammatory chemokine output through distinct mechanisms; they have not all been shown to regulate proximal IFN-γ signaling. Recognition can be impaired by coordinated HLA-I/APM downregulation, BCG-associated HLA-I loss, or TAP1 O-GlcNAcylation. In selected urothelial carcinoma models, PIK3CA mutation supports an immune-stimulation-responsive STAT1–IRF1–NLRC5–MHC-I program. CIITA/MHC-II and prolonged interferon states illustrate the context dependence of tumor-cell immune responses. Dashed connections indicate associations or conceptual links for which direct IFN-γ-dependent causality has not been established.

## Signal competence: impaired tumor-cell responsiveness to IFN-γ

The IFNGR–JAK–STAT1 pathway is the proximal route by which a malignant cell responds to IFN-γ. Defects at this level can affect several downstream responses at the same time.

Evidence in human urothelial cells comes from partial JAK1 deficiency. After IFN-γ stimulation, JAK1-deficient urothelial cells showed impaired STAT1 signaling and reduced induction of MHC-II, ICAM-1, and PD-L1. They were also resistant to IFN-γ-mediated apoptosis and less susceptible to lymphocyte-mediated killing (9). IFN-γ increased early apoptosis in control urothelial cells but produced no comparable increase in the JAK1-deficient cells (9).

The study defines a biological requirement for JAK1 in urothelial IFN-γ responsiveness, but its clinical scope is limited. The model arose from a rare germline partial JAK1 deficiency and does not show that somatic JAK1 loss commonly causes acquired ICI resistance in bladder cancer (9). Tumor-cell IFN-γ pathway loss and JAK1/2 loss-of-function mutations have been associated with checkpoint-blockade resistance in non-urothelial cancers (10, 11), but those observations provide cross-cancer context rather than bladder-specific evidence. Longitudinal urothelial datasets demonstrating treatment-emergent loss of JAK1, JAK2, or STAT1 remain limited.

AGO2 provides a different example of signal attenuation. In bladder cancer cells exposed to IFN-γ, AGO2 knockout increased STAT1 phosphorylation and nuclear localization together with CXCL9, ISG15, and HLA expression. When Stat1 was additionally deleted in Ago2-knockout MB49 tumors, the increase in CD8+ T-cell infiltration and cytotoxicity associated with Ago2 loss was reduced (12).

The regulatory circuit involves PTPN6 and miR-1246. IFN-γ increased AGO2 binding to PTPN6 mRNA, whereas AGO2 loss reduced PTPN6 transcript stability under IFN-γ stimulation and increased STAT1 activation. PTPN6 knockout also prevented AGO2 overexpression from suppressing STAT1, placing PTPN6 between AGO2 and STAT1 in the experimental system (12). IFN-γ increased miR-1246 abundance and its association with AGO2; inhibition of miR-1246 reduced AGO2–PTPN6 binding and PTPN6 mRNA stability and increased STAT1 activation (12).

This IFN-γ–miR-1246–AGO2–PTPN6–STAT1 feedback offers a route to functional desensitization without loss of IFNGR or JAK1. One mechanistic point remains unresolved. miR-1246 binds the PTPN6 coding sequence yet increases, rather than decreases, transcript stability, and the basis of this atypical miRNA effect has not been defined (12).

Structural pathway defects are therefore not the only route to reduced IFN-γ responsiveness. Even when proximal signaling is retained, the downstream transcriptional state may remain plastic. In muscle-invasive bladder cancer models, IFN-γ/JAK1/STAT1 signaling represses FOXA1 and promotes a basal transcriptional program, linking IFN-γ responsiveness to changes in tumor-cell state (13).

## Recruitment competence: uncoupling inflammatory signaling from T-cell access

CXCL9, CXCL10, and CXCL11 attract CXCR3-expressing effector lymphocytes and are repeatedly associated with ICI response in urothelial cancer (3, 4). IMvigor210 and GSE176307 also showed higher CXCL9/CXCL10 expression in atezolizumab-responsive tumors, although performance differed across datasets and did not establish either chemokine as a stand-alone predictive biomarker (14). Bladder cancer studies further show that tumor cells can suppress this chemotactic output without a demonstrated defect in proximal IFNGR–JAK–STAT signaling.

ZNF737 acts at the level of CXCL10 transcription. ZNF737 overexpression reduced CXCL10 promoter activity, and mutation of the implicated promoter sequence abolished this effect. CXCL10 neutralization reversed the increase in CD8+ T-cell migration produced by ZNF737 knockdown, whereas recombinant CXCL10 restored migration suppressed by ZNF737 overexpression (15). These experiments support a causal ZNF737–CXCL10 relationship in the tested models. High ZNF737 expression was also associated with poorer ICI outcomes in the clinical datasets analyzed by the investigators, but those data remain biomarker associations rather than proof that ZNF737 determines treatment response in patients (15).

HDAC10 reaches a similar immune output through a different mechanism. It deacetylates ILF3 at K342 and promotes WWP2-dependent ILF3 ubiquitination and degradation. ILF3 binds the CXCL9 3′-UTR; actinomycin-D experiments showed reduced CXCL9 mRNA stability after ILF3 depletion, and reporter assays supported binding of ILF3 to the predicted 3′-UTR region (16). The pathway is therefore supported as post-transcriptional regulation of CXCL9, not as a demonstrated defect in proximal IFN-γ signaling.

RRBP1 regulates CXCL10 through RNA stability. Genetic or pharmacological inhibition of RRBP1 increases cancer-cell CXCL10 secretion and promotes recruitment and effector activity of CXCR3+ CD8+ T cells. Blocking CXCL10 or CXCR3 only partially reverses this phenotype, indicating that the CXCL10–CXCR3 axis accounts for an important, but not exclusive, component of RRBP1-associated immune regulation (17). *In vivo*, RRBP1 inhibition improved anti-PD-L1 tumor control and was accompanied by increased CXCR3+, IFN-γ+, and GZMB+ CD8+ T-cell infiltration (17).

Other tumor-cell factors are associated with broader inflammatory outputs. S100A5 knockdown increased CCL2, CCL3, CCL4, CCL5, CXCL9, and CXCL10 secretion and CD8+ T-cell migration in T24 and 5637 cells (18). LRFN2 depletion similarly altered CCL2/3/4/5 and CXCL9/10 expression; in mouse tumors, LRFN2 knockdown increased CD8+ T-cell infiltration and enhanced anti-PD-1 activity (19). Neither study establishes direct regulation of proximal IFN-γ signaling. The HDAC inhibitor CM-1758 also altered PD-L1 regulation and increased CD8+ T-cell infiltration in experimental bladder cancer models (20).

A tumor cell can thus retain IFNGR–STAT1 activity while producing too little CXCL9 or CXCL10 to support effector-cell trafficking. We use the term chemokine-output uncoupling for this separation between inflammatory sensing and chemokine execution. It is a functional description and does not imply that all of the regulators above are direct components of the IFN-γ pathway.

Tumor-cell chemokine output is only one determinant of T-cell access. In immune-excluded metastatic urothelial tumors, fibroblast TGF-β activity was associated with non-response, and combined TGF-β/PD-L1 blockade in an experimental model altered T-cell positioning within the tumor bed (7). Chemokine insufficiency and stromal exclusion can therefore produce similar spatial patterns through different mechanisms.

## Recognition competence: preserving tumor antigen presentation

Effector cells that enter a tumor still require recognizable targets. For CD8+ T cells, recognition depends on the processing of intracellular antigens and presentation of peptide–HLA-I complexes at the malignant-cell surface.

Human bladder tumors can lose several components of this machinery simultaneously. In a series of 11 HLA-I-negative and 10 HLA-I-positive tumors, the HLA-I heavy chain, β2-microglobulin, TAP1, TAP2, LMP2, LMP7, and tapasin were lower in the HLA-I-negative group, while no β2-microglobulin coding mutations were found in the 11 negative tumors (21). The data support coordinated transcriptional suppression in that cohort rather than a dominant role for β2-microglobulin mutation. Preserved tumor-cell HLA-I expression was also associated with better recurrence-free survival after BCG in a small observational study (22), and a later pathological series linked altered HLA-I expression with T-cell exclusion and FAP-rich stromal changes (23).

BCG treatment provides an example of dynamic HLA-I regulation. Among 27 patients who relapsed after BCG, 13 tumors showed post-treatment HLA-I downregulation, whereas 14 maintained or increased HLA-I. Distant metastases subsequently developed in 9 of 13 patients in the HLA-I-downregulated group and in 2 of 14 patients in the HLA-I-maintained/upregulated group. The cohort was retrospective, and the clinical relationship was treated as correlative by the investigators (24). These findings describe heterogeneity among BCG-relapsed tumors and should not be extrapolated to all BCG-treated patients.

Live BCG exposure reduced surface HLA-I in a subset of bladder cancer cells, including fresh human tumor preparations and established cell lines (24). The phenotype was accompanied by altered autophagic flux. The experiments support a possible contribution of autophagy to BCG-associated HLA-I downregulation, but they do not define a complete causal pathway in which autophagic disruption alone accounts for the effect (24).

A more directly defined post-translational mechanism involves TAP1 O-GlcNAcylation. O-GlcNAc modification of TAP1 disrupted its interaction with MHC-I and promoted autophagic–lysosomal MHC-I degradation, reducing antigen presentation and CD8+ T-cell cytotoxicity (25). Ser63 was identified as an O-GlcNAcylated TAP1 site and linked to TAP1/HLA-A interaction and T-cell cytotoxicity (25). Loss of classical HLA-I does not eliminate every cytotoxic route: NKG2A+ CD8+ T cells can display innate-like activity against HLA-ABC-deficient bladder cancer cells, while HLA-E provides an inhibitory counter-signal (26). This axis is distinct from restoration of classical peptide–HLA-I recognition.

Recognition competence is not uniformly reduced. In experimental urothelial carcinoma models, oncogenic PIK3CA supported an immune-stimulation-responsive STAT1–IRF1–NLRC5–MHC-I program. PBMC co-culture increased IRF1 activation and antigen-presentation machinery more strongly in PIK3CA-mutant or PIK3CA-overexpressing cells, whereas PIK3CA depletion attenuated PBMC-induced STAT1 phosphorylation, IRF1, and MHC-I expression (27). PIK3CA loss also reduced MHC-I/B2M and weakened anti-PD-L1 activity *in vivo* (27).

The nature of the stimulus is important when interpreting these experiments. The principal mechanistic assays used PBMC co-culture rather than isolated IFN-γ. PIK3CA is therefore better described as a context-dependent regulator of an immune-responsive STAT1–IRF1–NLRC5 program in urothelial carcinoma than as an established direct activator of IFN-γ signaling.

Recruitment and recognition address different biological requirements. Effector lymphocytes must reach malignant cells, and the tumor cells must preserve sufficient antigen-processing machinery for productive T-cell recognition once contact occurs.

## Discussion: context, therapeutic modality, and clinical implications

Tumor-cell responsiveness to IFN-γ cannot be summarized on a simple high-versus-low scale. Different downstream programs have different biological consequences, and their relevance depends on therapeutic context.

CIITA illustrates this distinction in BCG-treated bladder cancer. IFN-γ activates CIITA through STAT1-dependent transcription, and CIITA deletion in MB49 tumors impaired BCG-mediated tumor elimination (28). Deletion of MHC-II itself did not reproduce this phenotype, and H2-DM was likewise dispensable (28). The data point to a BCG-relevant CIITA-dependent program broader than canonical MHC-II antigen presentation. In the same setting, CIITA was not required for response to PD-1 blockade (28), indicating that BCG and checkpoint inhibition can depend on non-identical tumor-cell programs.

Tumor-cell MHC-II expression requires similarly cautious interpretation. Single-cell and spatial analyses of urothelial carcinoma identified MHC-II+ malignant cells with increased JAK/STAT activity, STAT1/IRF1 activity, and CIITA expression. IFN-γ directly induced HLA-DRA in T24 and 5637 cells, and ruxolitinib suppressed the associated JAK1/STAT1 activation and HLA-DRA induction (29). These malignant cells lacked CD86 and should not be treated as conventional professional antigen-presenting cells (29).

Spatial analyses predicted colocalization of MHC-II+ malignant cells with IFNG-expressing CD8+ T cells, TNFRSF9+ Tregs, and SPP1+ macrophages (29). Several ligand–receptor interactions were inferred computationally rather than demonstrated functionally, and the investigators noted that most functional experiments were performed *in vitro* and that the predicted cellular interactions required *in vivo* validation (29). Current evidence therefore supports an IFN-γ-responsive MHC-II+ malignant-cell state within an exhaustion-enriched immune context, but it does not show that tumor-cell MHC-II causes CD8+ T-cell exhaustion. In FGFR3-activated NMIBC models, FGFR inhibition also altered the fate of IFN-γ-induced PD-L1 through autophagic degradation (30).

Signaling duration also matters. Across tumor types, IFN-γ has both immune-stimulatory and adaptive inhibitory effects (31, 32). In experimental cancer systems, prolonged type I and type II interferon signaling can maintain multigenic resistance programs, and chronic IFN-γ exposure can produce STAT1-associated epigenomic remodeling (33). Comparable longitudinal evidence has not established chronic IFN signaling as a recurrent mechanism of clinical ICI resistance in bladder cancer. These cross-cancer findings therefore caution against equating restored IFN-γ responsiveness with greater IFN-γ exposure.

Clinical resistance is similarly heterogeneous. Paired PURE-01 samples showed distinct post-pembrolizumab transcriptional and microenvironmental states rather than one resistance phenotype (34), and relapse-focused analyses identified immune-associated genes including CXCL9 and CXCL11 among transcripts linked to recurrence (35). ABACUS associated baseline immune activation and treatment-induced remodeling with response to neoadjuvant atezolizumab, while stromal programs characterized a subset of resistant tumors (36). BCG-treated NMIBC also shows adaptive immune remodeling in paired specimens (37). Other studies capture heterogeneity through T-cell-to-stroma enrichment (6), different cellular sources detected by PD-L1 assays (38), site-specific immune environments in multiregional metastatic disease (39), and distinct transcriptional states in large trial-integrated urothelial carcinoma datasets (40).

No study cited here validates a clinical “tumor-intrinsic IFN-γ competence score.” That concept remains a hypothesis. Testing it would require malignant-cell-resolved measurements of proximal IFN-γ signaling, tumor-derived CXCL9/CXCL10 output, and HLA-I/APM integrity in paired pretreatment, on-treatment, and resistant specimens. Spatial information would also be needed because chemokine output cannot overcome every stromal barrier to lymphocyte access (6, 7). The context-dependent performance of T-cell-inflamed signatures, CXCL9/CXCL10, and PD-L1 argues against reliance on a single analyte (5, 14, 38).

The therapeutic implications are likewise mechanism-specific. A tumor with impaired IFNGR–STAT1 responsiveness differs biologically from one with preserved proximal signaling but low CXCL9/CXCL10 output, and both differ from a tumor that recruits T cells yet loses HLA-I presentation. The evidence reviewed here does not support a single “IFN-γ-restoring” intervention for all three situations.

A more useful clinical question may therefore be whether malignant urothelial cells can execute the immune functions required for effector-cell access and tumor recognition, rather than simply whether IFN-γ is present within the tumor. This remains a testable proposal, not a validated biomarker model. Its purpose is to separate mechanistically distinct forms of immune failure that may look similar in bulk inflammatory profiles. The tumor-intrinsic regulators and response states discussed in this Review, together with their functional level, relationship to IFN-γ, experimentally supported immune outputs, and key evidence boundaries, are summarized in **Table 1**.

**Table 1:** Tumor-intrinsic regulators of IFN-γ-associated immune execution in bladder cancer.

Regulator or state	Functional level	Relationship to IFN-γ	Main experimentally supported output	Immune consequence	Evidence boundary
IFNGR loss	Signal sensing	Direct	Loss of tumor-cell IFN-γ response	Impaired BCG-mediated tumor control	Demonstrated in MB49 BCG model
JAK1 deficiency	Signal transduction	Direct	Reduced STAT1/IRF1 response; impaired IFN-γ-induced apoptosis	Reduced lymphocyte-mediated killing	Human urothelial-cell model; not established as common acquired ICI resistance
miR-1246–AGO2–PTPN6	Signal regulation	Direct	PTPN6 stability↑; pSTAT1↓	Reduced CD8+ T-cell immunity	Mechanistically supported; basis of atypical PTPN6 RNA stabilization remains unresolved
ZNF737	Chemokine transcription	Downstream output	CXCL10 transcription↓	Reduced CD8+ T-cell chemotaxis	No evidence that ZNF737 inhibits proximal IFNGR signaling
HDAC10–ILF3–WWP2	Post-transcriptional chemokine regulation	Downstream output	CXCL9 mRNA stability↓	Reduced CD8+ T-cell recruitment/activation	Non-histone mechanism; not a demonstrated proximal IFN-γ defect
RRBP1	RNA stability	Downstream output	CXCL10 availability↓	Reduced CXCR3+ CD8+ T-cell recruitment/function	CXCL10–CXCR3 explains part, not necessarily all, of the phenotype
S100A5/LRFN2	Broad inflammatory output	No direct proximal link established	CXCL9/10 and other inflammatory chemokines↓	Reduced CD8+ T-cell access/function	Convergent supporting evidence
Coordinated HLA-I/APM downregulation	Antigen presentation	Some regulatory defects are IFN-γ reversible	HLA-I, B2M, TAP/LMP/tapasin↓	Reduced tumor recognition	Based partly on small human bladder cancer series
BCG-associated HLA-I loss	Dynamic recognition state	Treatment-context dependent	Surface HLA-I↓	Distinct immune phenotype in BCG-relapsed tumors	Patient outcome data are retrospective and correlative
TAP1 O-GlcNAcylation	Post-translational antigen presentation	No direct IFN-γ link established	TAP1–MHC-I interaction↓; MHC-I degradation↑	Reduced CD8+ T-cell cytotoxicity	Mechanistic preclinical evidence
PIK3CA-associated immune-responsive state	Recognition competence	Intersects with STAT1; not proven IFN-γ-specific	IRF1/NLRC5/MHC-I↑ after immune stimulation	Increased antigen presentation and anti-PD-L1 activity	Key experiments used PBMC stimulation
CIITA	Therapy-dependent output	Directly IFN-γ inducible	Broad CIITA-dependent transcriptional program	Required for BCG efficacy in MB49	MHC-II itself dispensable; CIITA not required for anti-PD-1 in same model
MHC-II+ malignant-cell state	Context-dependent output	IFN-γ inducible	HLA-DRA/MHC-II program↑	Associated with a complex immune ecosystem	Spatial interactions largely associative/computational

## Conclusions

Current bladder cancer evidence identifies several tumor-intrinsic points at which inflammatory immunity can fail. Loss or attenuation of IFNGR–JAK–STAT1 responsiveness can reduce tumor-cell sensitivity to IFN-γ. Other mechanisms have not been shown to disrupt proximal signaling but can suppress CXCL9/CXCL10-associated T-cell recruitment. HLA-I and antigen-processing machinery can also be altered transcriptionally, during therapy, or through post-translational modification.

These mechanisms should not be treated as a single linear IFN-γ resistance pathway. Their shared feature is functional: each can weaken the conversion of an immune-inflamed environment into tumor-cell elimination.

The same evidence argues against assuming that every IFN-γ-responsive state is beneficial. CIITA is required differently in BCG and PD-1 blockade models (28), tumor-cell MHC-II occurs within heterogeneous immune ecosystems (29), and prolonged interferon signaling can support adaptive resistance in other cancer systems (31–33). Treatment modality, signaling duration, and tissue context therefore remain important to interpretation.

Prospective longitudinal studies that distinguish malignant-cell responses from immune and stromal signals are needed to determine whether defects in signaling, recruitment, and recognition define reproducible treatment-resistance states. Until such validation is available, the three proposed levels are best used to organize current mechanistic evidence and to formulate testable biomarker and combination-treatment hypotheses.
